# Helcococcus kunzii as a rare etiological agent of spondylodiscitis partially managed with oral antibiotic therapy in a patient with diabetes mellitus: a case report

**DOI:** 10.1186/s12879-026-14341-z

**Published:** 2026-09-18

**Authors:** Karen Velázquez-Leonel, Christian Bepperling, Thomas Regnath, Farzam Vazifehdan, Edgar Santos

**Affiliations:** 1 Spine Center Stuttgart, Paulinenhilfe, Diakonie-Klinikum Stuttgart, Rosenbergstraße 38, 70176 Stuttgart, Germany; 2 Carl von Ossietzky Universität Oldenburg, Marienstraße 11, 26121 Oldenburg, Germany; 3 Laboratory Prof. G. Enders and colleagues, Rosenbergstraße 85, 70193 Stuttgart, Germany

**Keywords:** Spondylodiscitis, *Helcococcus kunzii*, MALDI-TOF MS, Diabetes mellitus, Case report

## Abstract

**Background:**

Previous studies have reported *Helcococcus kunzii* infection in a range of clinical conditions, including infective endocarditis, umbilical abscesses, bacteremia, diabetic foot infections, and prosthetic joint infections. Diabetes mellitus has been identified as a predisposing condition for infections caused by this pathogen. We report the first documented case of spondylodiscitis caused by *Helcococcus kunzii.*

**Case presentation:**

We describe a case of spondylodiscitis caused by *Helcococcus kunzii* in a 62-year-old man with long-standing type 1 diabetes mellitus who presented to the emergency department with severe, immobilizing lumbar pain. Clinical diagnosis of spondylodiscitis was established by magnetic resonance imaging (MRI). *Helcococcus kunzii* was isolated from four separate blood culture sets. During surgical intervention, two swabs and two tissue samples were collected. *H. kunzii* was identified in all specimens using MALDI-TOF mass spectrometry (MALDI-TOF MS) with log scores ranging from 2.30 to 2.52. The patient received intravenous β-lactam antibiotics for ten days, followed by 13 weeks of oral β-lactam antibiotics. Follow-up MRI performed before discharge showed no further evidence of spondylodiscitis. At the 3-month follow-up visit, the patient demonstrated clinical improvement.

**Conclusions:**

This case demonstrates that *H. kunzii* should be considered a potential etiological agent of pyogenic spondylodiscitis, particularly in patients with diabetes mellitus and associated comorbidities that may compromise skin barrier integrity. MALDI-TOF MS is essential for accurate identification of this organism and should be considered in cases of atypical spondylodiscitis.

## Background

*Helcococcus kunzii* is a Gram-positive, catalase-negative, facultatively anaerobic, non-motile, and non-sporulating bacterium that was first recognized as a commensal organism of human skin, particularly of the lower extremities. Because infections have been reported predominantly in immunocompromised individuals, especially males with diabetes mellitus, it was initially considered an opportunistic pathogen [1, 2]. However, cases have also been described in immunocompetent patients [3].

*H. kunzii* has been implicated in a wide range of infections, including central nervous system infections, infective endocarditis, umbilical abscesses, bacteremia, diabetic foot infections, and prosthetic joint infections [3–7].

Traditional biochemical identification methods often fail to accurately identify *H. kunzii*, leading to misidentification as Aerococcus species. Matrix-assisted laser desorption/ionization–time of flight mass spectrometry (MALDI-TOF MS) has proven to be a reliable method for precise identification, with log scores > 2.0 supporting species-level identification [8].

Currently, no standardized antibiotic treatment guidelines have been established for *H. kunzii* infections. Nevertheless, antimicrobial susceptibility studies indicate that most isolates are generally susceptible to β-lactam antibiotics [9].

We report the first documented case of spondylodiscitis caused by *Helcococcus kunzii.*

## Case presentation

A 62-year-old male patient presented to the emergency department with severe, immobilizing lumbar pain. His medical history was notable for an L2 vertebral endplate compression fracture diagnosed one year prior, managed conservatively with ibuprofen 400 mg every 12 h. Two weeks before the current presentation, the patient reported a significant intensification of pain; despite two epidural corticosteroid injections, symptomatic relief remained minimal. In the two days preceding admission, pain escalated to the point of complete bed confinement. The patient denied associated fever. Due to progressive symptom worsening, he self-administered two doses of diclofenac 75 mg one day before presentation, without relief.

The patient’s past medical history was significant for type 1 diabetes mellitus, diagnosed at age 30, with a recent glycated hemoglobin (HbA1c) of 5.3%. Insulin therapy consisted of a basal-bolus regimen with continuous glucose monitoring, achieving adequate glycemic control, with all fasting blood glucose values in the week preceding presentation below 180 mg/dL.

Additional comorbidities included systemic arterial hypertension managed with a standard three-drug regimen; class II obesity per the World Health Organization (WHO) BMI classification (BMI 38.7 kg/m²); and stage II chronic venous insufficiency per the Widmer classification. No known drug allergies were reported.

**Fig. 1:**
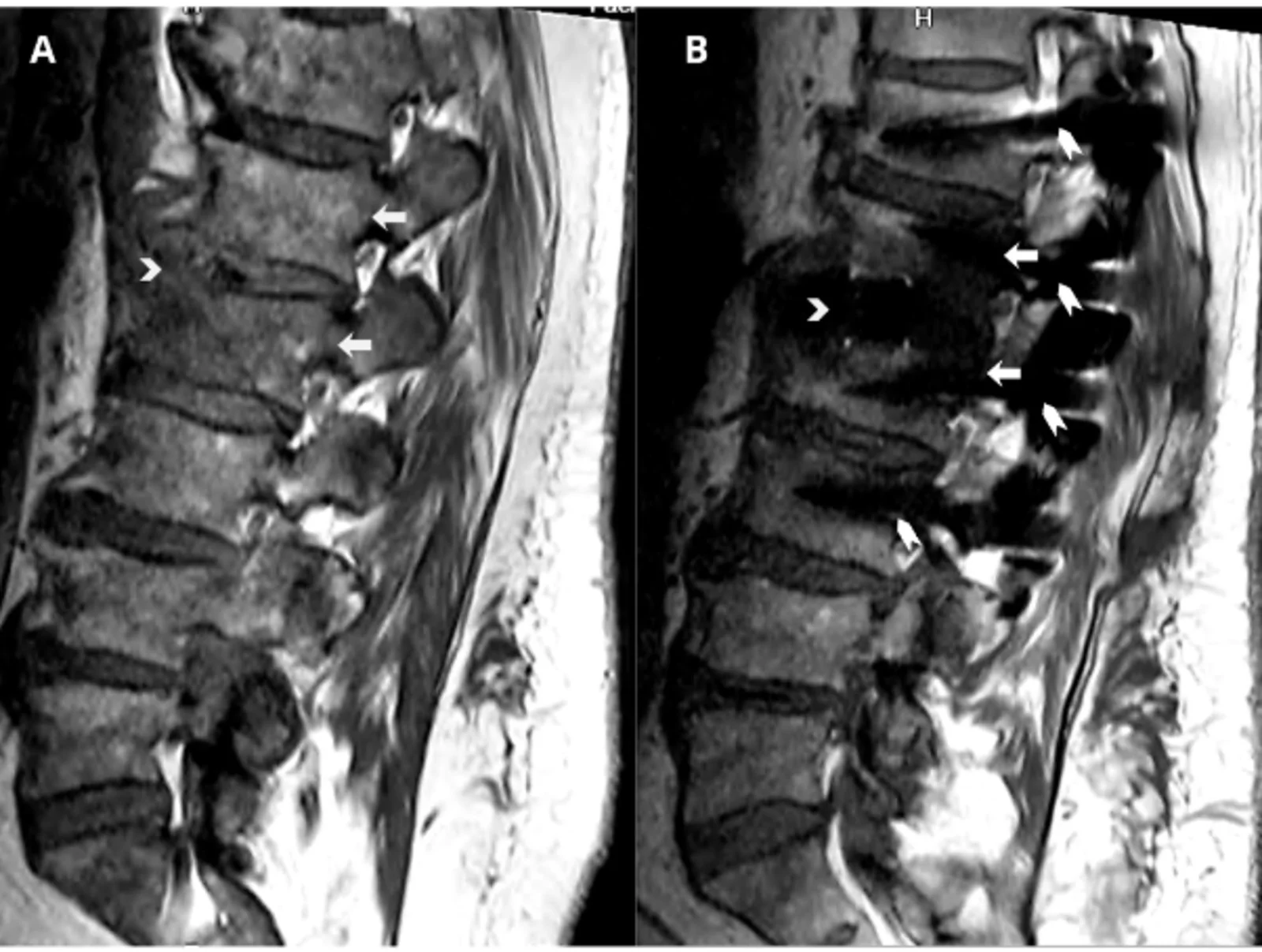
(**A**) MRI provided by the patient at admission. (**B**) Follow-up MRI performed before discharge, after the surgical procedure. Short arrowheads indicate changes in the intervertebral space associated with the infection. Long arrows indicate vertebrae L1–L2. Long arrowheads indicate the osteosynthesis material

Given the progressive worsening of symptoms, the patient sought evaluation by his treating physician, who requested a lumbar magnetic resonance imaging (MRI) study. The MRI protocol included sagittal T1- and T2-weighted turbo spin-echo sequences, axial T2-weighted turbo spin-echo sequences from T12 to S1, and a coronal T1-weighted short tau inversion recovery (STIR) sequence. Imaging demonstrated progressive destruction of the L1–L2 intervertebral disc space with associated bone marrow edema, raising suspicion for an infectious etiology (Fig. 1). Based on these findings, the patient was referred to our institution for further evaluation and management.

On admission, the patient reported constipation, which he attributed to reduced oral intake secondary to pain severity and limited mobility; he reported ingesting approximately 500 mL of water daily during the preceding week. Urinary function was preserved, with no evidence of urinary retention or incontinence. No signs of cauda equina syndrome or spinal cord dysfunction were identified.

Neurological assessment revealed motor strength of 5/5 in all lower-extremity myotomes bilaterally per the Medical Research Council (MRC) scale. Patellar tendon reflexes were present bilaterally; Achilles tendon reflexes were absent bilaterally. Sensory examination was unremarkable. Despite intact motor strength and sensation, the patient was non-ambulatory due to pain. Vital signs were within normal limits; no fever, hypotension, or altered mental status were detected.

Dermatological and podiatric examination of the lower extremities revealed bilateral stasis dermatitis without ulceration. Dorsalis pedis pulses were palpable bilaterally. The patient was wearing compression stockings consistent with his known chronic venous insufficiency. Examination of the right foot demonstrated a pronounced claw toe deformity of the second and third digits with marked hyperkeratosis and a superficial wound classified as Wagner grade 1, Armstrong stage A. Interdigital tinea pedis was also noted.

Admission laboratory investigations revealed leukocytosis (11,500 cells/µL) with neutrophilia (7.9 × 10³/µL; 80.8%), a C-reactive protein (CRP) of 7.7 mg/dL, an erythrocyte sedimentation rate (ESR) of 50 mm/h, a serum creatinine of 4.3 mg/dL, and a blood urea nitrogen (BUN) of 175 mg/dL.

Given the severity of renal impairment, a native (non-contrast) spiral CT of the lumbar spine with multiplanar reconstructions in bone window was performed instead of contrast-enhanced MRI, and a nephrology consultation was requested.

**Fig. 2:**
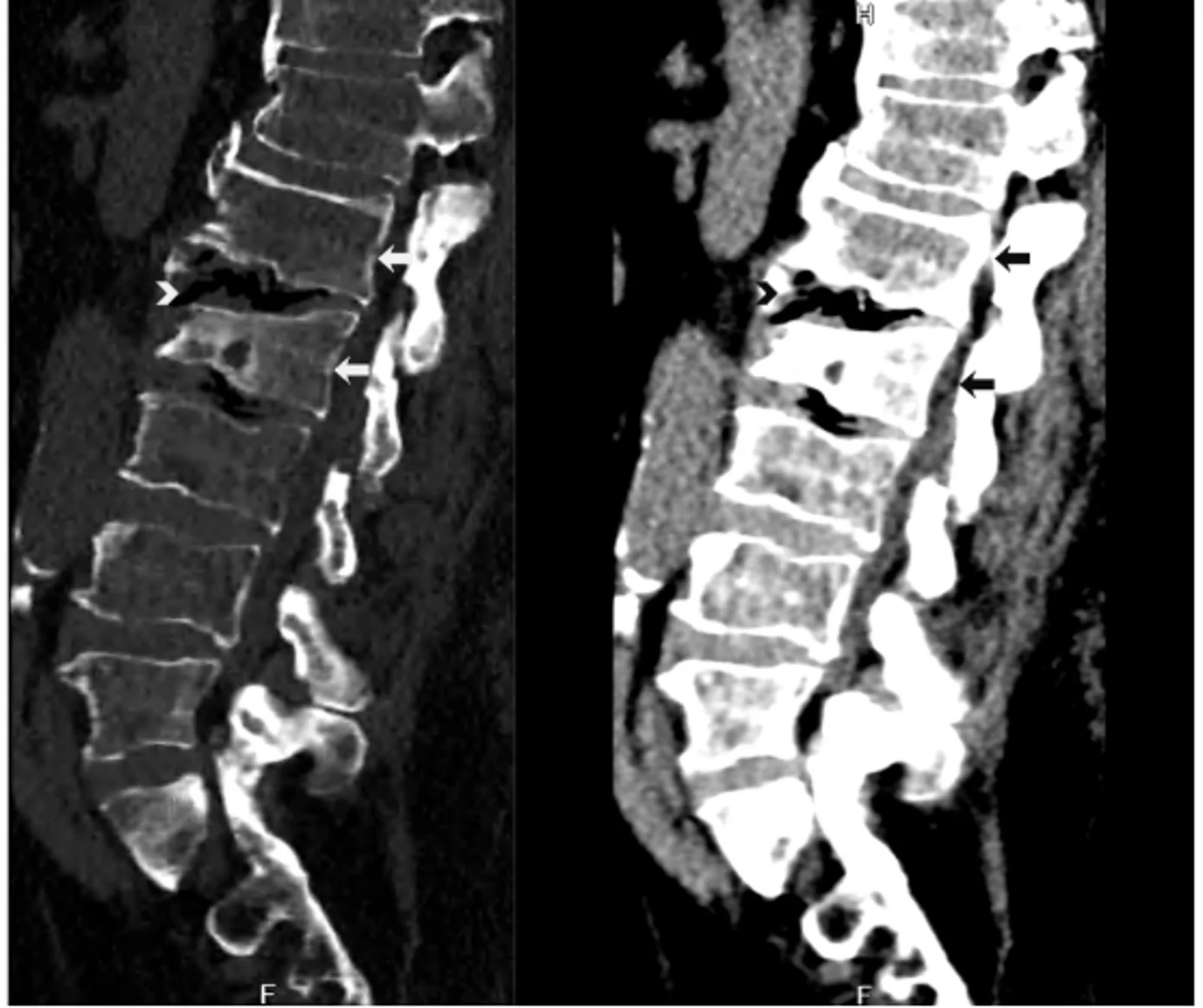
(**A**) CT performed at admission. Arrowhead indicates vacuum phenomenon in the disc space. Long arrows indicate vertebrae L1 and L2

CT imaging demonstrated reduced anterior endplate height at L2 with cortical thinning and disruption, a fractured right-sided anterior osteophyte, and a significant vacuum phenomenon within the L1–L2 disc space. The disc space contained fluid-isodense material with fat extending retrocrurally on the right side, along with air inclusions measuring 34 × 17 × 85 mm. An intraosseous disc herniation (Schmorl’s node) was identified at the L2 inferior endplate. Additional findings included multisegmental degenerative changes and osteochondrosis. (Fig. 2).

The patient was subsequently diagnosed with acute kidney injury (AKI) classified as Stage 3 per the Acute Kidney Injury Network (AKIN) criteria, attributed to prolonged NSAID use. NSAIDs were promptly discontinued, and supportive management with intravenous hydration and analgesic adjustment was initiated. Thromboprophylaxis was initiated with subcutaneous unfractionated heparin (UFH).

As part of the diagnostic investigations, four blood culture sets were obtained, two aerobic and two anaerobic pairs. Results were available after five days of incubation; all anaerobic specimens yielded growth of *Helcococcus kunzii.*

**Fig. 3:**
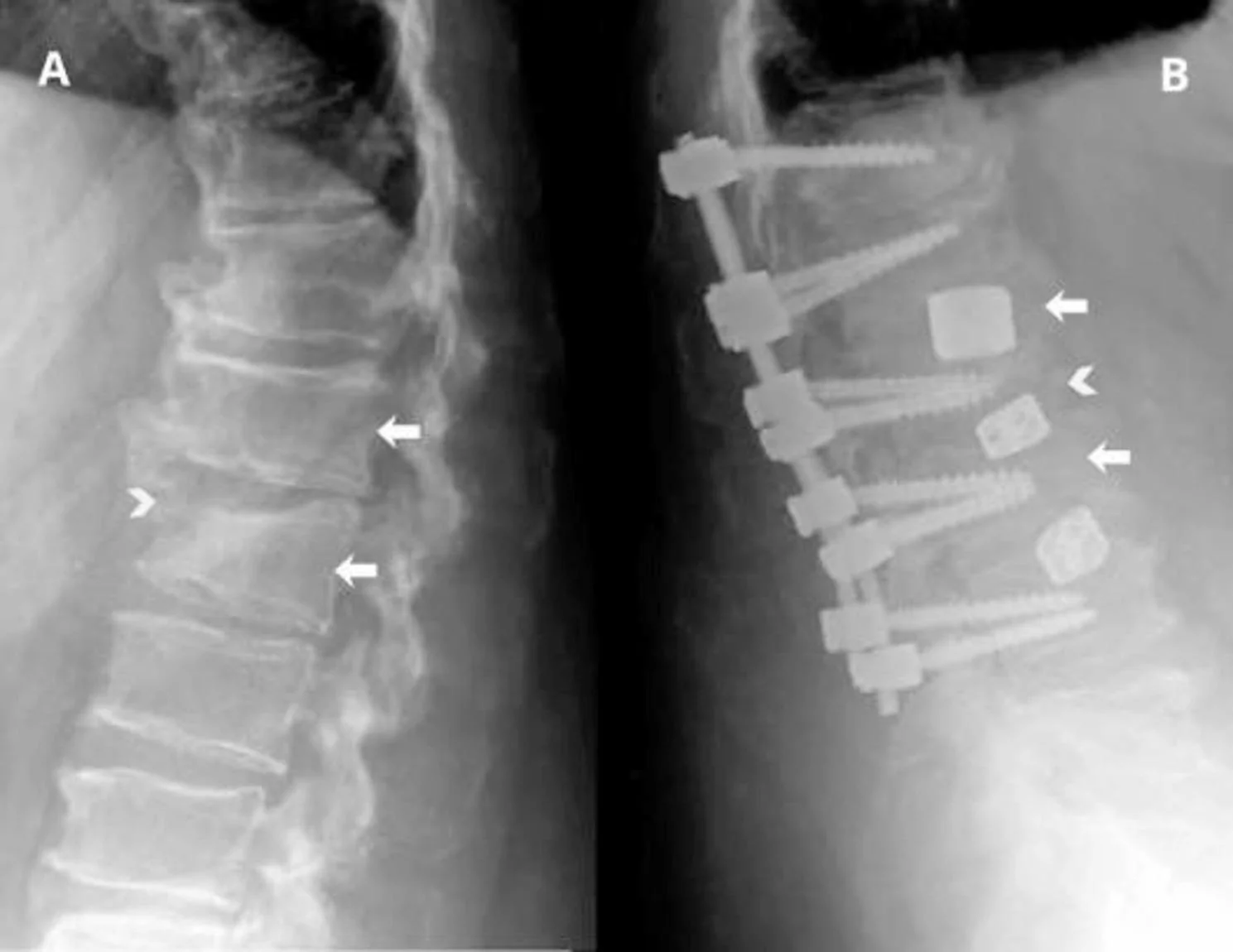
Lateral X-ray of the lumbar spine. (**A**) Performed at admission. (**B**) Postsurgical changes. Arrowhead indicates disc space. Long arrows indicate vertebrae L1 and L2

Following multidisciplinary assessment, surgical management was indicated given refractory pain with significant functional impairment, failure of conservative measures, progressive destructive disease course, and incipient segmental kyphosis. Six days after admission, and one day following the availability of blood culture results, the patient underwent combined minimally invasive dorsoventral thoracolumbar spinal fusion from T12 to L3. Perioperative antibiotic prophylaxis was administered with cefazolin 2 g intravenously prior to incision. The procedure consisted of navigated percutaneous posterior pedicle screw instrumentation (Voyager System; Medtronic, Dublin, Ireland) and extreme lateral interbody fusion (XLIF) at the L1–L2 level (NuVasive, San Diego, CA, USA) (Fig. 3). Intraoperative findings were consistent with an infected hematoma and abscess arising from erosive endplate destruction, with an adjacent fluid collection. The procedure was completed without intraoperative complications.

During the surgical procedure, two swabs and two tissue samples were collected from the L1–L2 intervertebral disc space; all specimens yielded growth of *Helcococcus kunzii* (no aspiration had been performed beforehand). Additionally, *Cutibacterium acnes* was isolated from two of the four samples (one swab and one tissue sample). All *H. kunzii* isolates were identified by MALDI-TOF MS; log scores ranged from 2.30 to 2.52, considered indicative of reliable species-level identification. Histopathological examination with hematoxylin and eosin (H&E), Elastica van Gieson (EVG), iron staining, and CD15 immunostaining demonstrated focal, moderate, florid phlegmonous inflammation in portions of the nucleus pulposus, consistent with spondylodiscitis.

Antibiotic therapy was initiated with a regimen of ampicillin/sulbactam 3 g and penicillin G at a dose of 10 million IU administered by intravenous infusion.

Following seven days of subcutaneous UFH therapy, the platelet count declined to 20,000/µL, raising suspicion for heparin-induced thrombocytopenia type II (HIT), supported by a 4Ts score of 6 (high pretest probability) and a positive PF4-ELISA (HIPA assay negative). UFH was discontinued and fondaparinux was initiated; the platelet count subsequently recovered to 124 × 10³/µL by Day 16.

Antimicrobial susceptibility testing (AST) of the blood culture isolate was available four days postoperatively. Because no official European Committee on Antimicrobial Susceptibility Testing (EUCAST) breakpoints have been established for *Helcococcus kunzii*, susceptibility was interpreted using breakpoints defined for *Streptococcus* species, in accordance with EUCAST guidelines and justified by the phylogenetic relationship between these genera. Susceptibility was determined by gradient diffusion (E-test); broth microdilution and disk/agar diffusion were not performed. The isolate was susceptible to penicillin G, ampicillin/sulbactam, and rifampicin, and resistant to ciprofloxacin, trimethoprim/sulfamethoxazole, and clindamycin. Because resistance was determined from MIC values obtained by E-test rather than by an induction (D-) test, a constitutive versus inducible mechanism of clindamycin resistance could not be formally distinguished. Minimum inhibitory concentrations are provided in Table 1. The ongoing antibiotic regimen was consistent with these susceptibility results and was continued without modification.

**Table 1:** Minimum inhibitory concentrations (MIC) of the *Helcococcus kunzii* isolate

Antimicrobial Agent	MIC (mg/l)	Interpretation
Penicillin G	0.016	susceptible
Ampicillin/Sulbactam	< 0.16	susceptible
Rifampicin	< 0.16	susceptible
Ciprofloxacin	1.5	resistant
Trimethoprim/Sulfamethoxazole	> 32	resistant
Clindamycin	> 256	resistant

Given previously reported cases of *Helcococcus kunzii* as a causative agent of infective endocarditis, a transesophageal echocardiogram was performed. No evidence of valvular vegetations or endocarditis was identified.

**Fig. 4:**
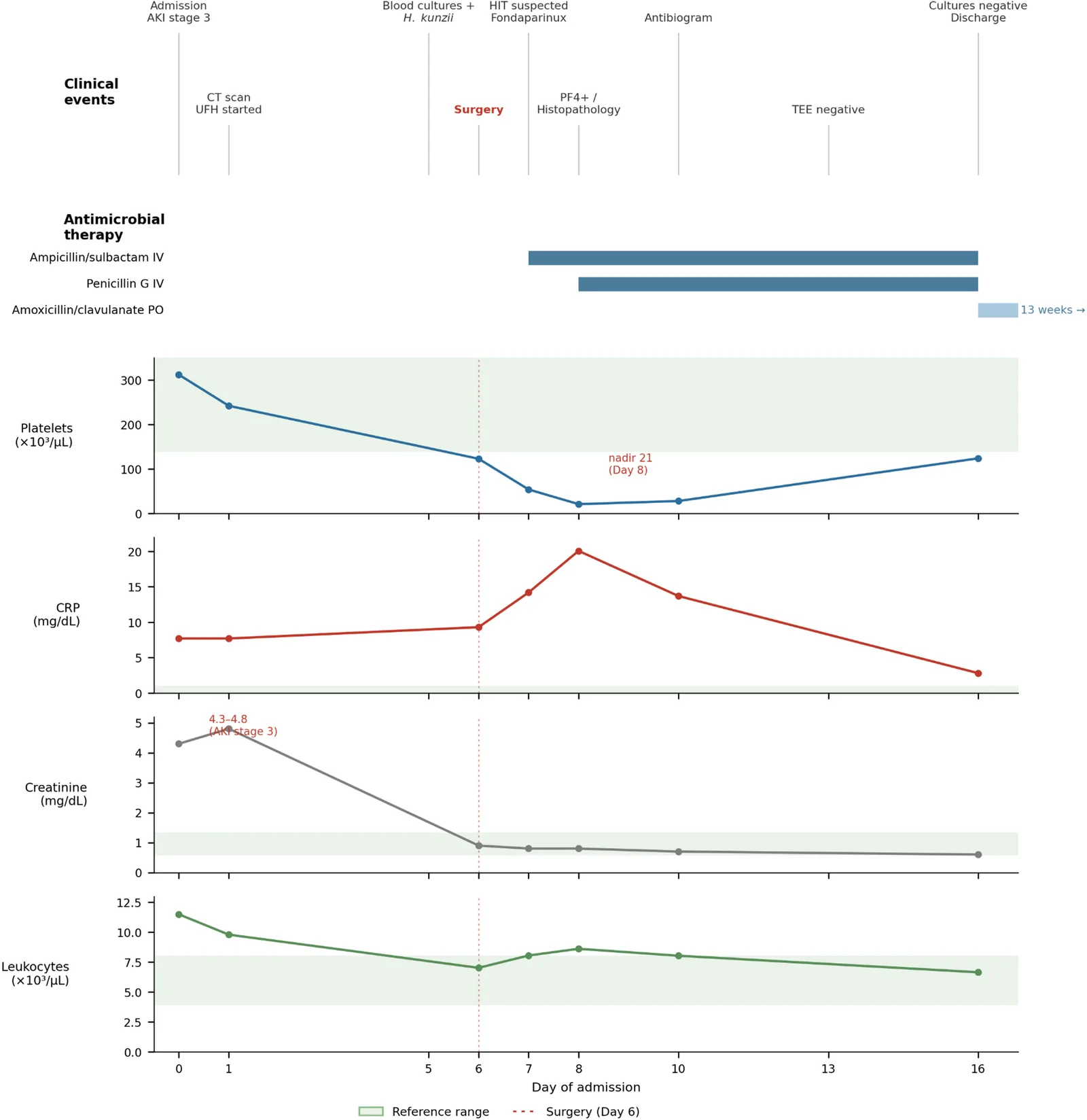
Integrated clinical timeline. Top: key clinical and diagnostic events relative to day of admission. Middle: duration of intravenous and oral antimicrobial therapy. Bottom: trends in platelet count, C-reactive protein, creatinine, and leukocyte count, with reference ranges shaded in green. The vertical dashed line indicates the day of surgical intervention (Day 6). The platelet nadir (21 × 10³/µL) on Day 8 coincided with the diagnosis of heparin-induced thrombocytopenia and the switch from unfractionated heparin to fondaparinux

The evolution of the patient’s laboratory parameters relative to the institutional reference values is summarized in Fig. 4.

The patient received intravenous antibiotic therapy for ten days. No sensorimotor deficits were observed, and a pain-adapted reduction in analgesic therapy was successfully achieved. The patient underwent physiotherapy to aid mobilization and was able to ambulate with a walker prior to discharge. Follow-up blood cultures obtained on Day 15 of admission (Day 9 of antibiotic therapy) showed no microbial growth in either aerobic or anaerobic bottles.

Before discharge, a follow-up MRI of the entire spine was performed, demonstrating no evidence of spondylodiscitis at additional levels in the thoracic or cervical spine (Fig. 1). Following discharge, the patient continued antibiotic therapy with oral amoxicillin/clavulanic acid 875/125 mg every eight hours for 13 weeks.

## Discussion

This case represents, to our knowledge, the first documented instance of spondylodiscitis caused by *Helcococcus kunzii*, expanding the known spectrum of invasive infections attributable to this organism. With specific regard to bone and joint infections, Pérez et al. reported the case of a young patient with elevated inflammatory markers who developed two soft tissue masses 14 months after total knee replacement. During a two-stage exchange procedure, tissue samples were obtained and *H. kunzii* was identified using the API 20 STREP system. The authors postulated that, because the organism is mainly isolated from the lower extremities, contamination likely occurred during the initial surgery [3]. Furthermore, Li et al. reported the isolation of *H. kunzii* from a patient with a diabetic foot infection [7].

Given that *H. kunzii* has previously been isolated from lower extremity wounds, many of which were diabetic foot lesions [10], the portal of entry in the present case may have been the cutaneous wound on the right foot, classified as Wagner grade 1, Armstrong stage A, in a patient with concurrent interdigital tinea pedis.

By contrast, *H. kunzii* has not been reported in any series of epidural-injection-related spinal infections, which are overwhelmingly attributable to *S. aureus*, other Gram-positive cocci, Gram-negative organisms, or, in recognized outbreaks, *Serratia marcescens* from contaminated contrast vials or fungal contamination with *Exserohilum rostratum* from compounded steroid preparations. In both cases, the mechanism of infection is entirely inconsistent with a skin commensal such as *H. kunzii* [11]. Severe infection following single-shot epidural corticosteroid injection is, in any case, rare [11, 12].

Taken together, the organism-specific literature consistently links *H. kunzii* to lower-extremity skin and wound reservoirs rather than to invasive procedures, supporting the cutaneous foot wound—rather than the epidural corticosteroid injections—as the more biologically plausible portal of entry in this case, although a definitive causal relationship cannot be established from case-report-level evidence alone.

We propose the following sequence of events as the most likely pathogenic mechanism in this case. First, the integrity of the skin barrier was compromised by multiple concurrent conditions - interdigital tinea pedis, the Wagner grade 1 wound, and stasis dermatitis - each of which may have served as an independent portal of entry. Second, *H. kunzii* translocated from the affected skin or wound into the bloodstream. Third, this bacteremia seeded the vertebral column, with a predilection for the highly vascularized vertebral endplates and intervertebral disc space. Finally, this hematogenous seeding led to the establishment of spondylodiscitis within the disc space and adjacent vertebrae.

The isolation of *C. acnes* from two of four intraoperative specimens most likely reflects intraoperative skin contamination rather than a true co-pathogen. Growth was inconsistent across specimens—in contrast to the uniform recovery of *H. kunzii* from all four—and the acute pyogenic histopathological pattern observed is atypical for *C. acnes* spinal infection. *C. acnes* is a well-recognized contaminant in spinal surgery [13–16]. Nonetheless, a polymicrobial infection cannot be definitively excluded on microbiological grounds alone, and this is acknowledged as a limitation of the present case.

Because no specific treatment guidelines have been established for *H. kunzii*, susceptibility results were interpreted using EUCAST breakpoints established for *Streptococcus* species, as justified by the phylogenetic relationship between these genera [17]. In the present case, the patient responded favorably to β-lactam therapy, with negative follow-up cultures, findings consistent with previously published reports.

Although the use of a short intravenous course followed by 13 weeks of oral treatment may be considered atypical for pyogenic spondylodiscitis, this approach is supported by recent evidence on early oral switch strategies in bone and joint infections. The landmark OVIVA trial (2019) demonstrated that oral antibiotics were noninferior to intravenous therapy for bone and joint infections when used during the first six weeks of treatment, with oral therapy associated with shorter hospital stays and fewer complications [18]. A systematic review of 21 prospective controlled trials similarly found that oral antibiotics were at least as effective as intravenous-only therapy for osteomyelitis, with no studies demonstrating superiority of intravenous-only treatment [19]. Furthermore, Babouee et al. conducted a retrospective study of 61 patients with vertebral osteomyelitis and reported high cure rates with comparatively short intravenous courses and overall antibiotic durations, proposing that transitioning to an oral antibiotic regimen after two weeks of intravenous therapy may be safe, provided that CRP has decreased and any epidural or paravertebral abscesses of significant size have been drained [20].

It must be acknowledged, however, that the evidence base supporting this approach has important limitations. The OVIVA trial enrolled a heterogeneous population of bone and joint infections, in which patients undergoing surgery for discitis, spinal osteomyelitis, or epidural abscess (with or without débridement) comprised only approximately 3.7% of the total study population [18]. The study by Babouee et al. supporting early oral switch in vertebral osteomyelitis is retrospective in design, which limits the strength of its conclusions [20]. Regarding the total duration of 13 weeks of oral therapy, no published evidence specifically supports this duration for infections caused by *H. kunzii*, as no prospective studies or organism-specific treatment guidelines exist for this pathogen. This decision was therefore based on clinical judgment informed by two considerations: the limited published experience with *H. kunzii* as a causative agent of spondylodiscitis, and the presence of spinal implants (T12–L3 instrumented fusion), which is associated with an increased risk of implant-associated infection and generally warrants a more prolonged antibiotic course than uncomplicated spondylodiscitis managed without hardware.

This case has additional limitations. The antibiotic regimen, including its duration and route, was not guided by established evidence for this specific pathogen, and further prospective studies are needed to define optimal treatment strategies for spondylodiscitis caused by *H. kunzii* and other rare organisms. Serial ESR measurements were not obtained during the hospitalization, limiting the use of this marker for monitoring treatment response. Finally, histopathological images were not available for inclusion in the manuscript. Notwithstanding these limitations, we believe this case provides a valuable contribution to the understanding of the pathogenic potential of *H. kunzii* and to the clinical and microbiological approach to spondylodiscitis caused by rare organisms.

## Conclusions

We report the first documented case of spondylodiscitis caused by *Helcococcus kunzii*, expanding the known spectrum of invasive infections attributable to this organism. This case demonstrates that *H. kunzii* should be considered a potential etiological agent of pyogenic spondylodiscitis, particularly in patients with longstanding diabetes mellitus and associated comorbidities — including peripheral neuropathy, chronic venous insufficiency, and obesity — that may compromise skin barrier integrity and facilitate hematogenous dissemination from lower extremity lesions.

Because *H. kunzii* is difficult to identify using conventional biochemical methods and may be misidentified as Aerococcus or streptococcal species, molecular diagnostic tools such as MALDI-TOF MS should be considered for the analysis of blood cultures and intraoperative specimens in patients with atypical spondylodiscitis, particularly those with diabetes mellitus or other predisposing comorbidities. Accurate microbiological identification is essential to guide targeted antimicrobial therapy and to minimize the risk of serious complications, including spinal instability and neurological compromise.

Successful management in this case required a multidisciplinary approach combining surgical stabilization, targeted intravenous β-lactam therapy, and an extended course of oral amoxicillin-clavulanate guided by clinical response and microbiological clearance. Given the increasing number of reported *H. kunzii* infections and the high prevalence of chronic comorbidities among affected patients, continued surveillance and further research are warranted to better characterize the pathogenic mechanisms, antimicrobial resistance patterns, and optimal treatment strategies for this emerging pathogen.

## Data Availability

All data generated or analysed during this study are included in this published article.

## Abbreviations

4Ts: four T's scoring system (Thrombocytopenia, Timing, Thrombosis, oTher causes) for heparin-induced thrombocytopenia
AKI: Acute kidney injury
AKIN: Acute kidney injury network
AST: Antimicrobial susceptibility testing
BMI: Body mass index
BUN: Blood urea nitrogen
CD15: Cluster of differentiation 15
CGM: Continuous glucose monitoring
CRP: C-reactive protein
CT: Computed tomography
ESR: Erythrocyte sedimentation rate
EUCAST: European committee on antimicrobial susceptibility testing
EVG: Elastica van gieson
H&E: Hematoxylin and eosin
HbA1c: eosinHbA1c: glycated
HIPA: Heparin-induced platelet activation
HIT: Heparin-induced thrombocytopenia
IU: International units
IV: Intravenous
MALDI-TOF MS: Matrix-assisted laser desorption/ionization time-of-flight mass spectrometry
MIC: Minimum inhibitory concentration
MRC: Medical research council
MRI: Magnetic resonance imaging
NSAID: Nonsteroidal anti-inflammatory drug
PF4-ELISA: Platelet factor 4 enzyme-linked immunosorbent assay
STIR: Short tau inversion recovery
UFH: Unfractionated heparin
WHO: World health organization
XLIF: extreme lateral interbody fusion
